# Quantifying cervical spondylosis: reliability testing of a coherent CT-based scoring system

**DOI:** 10.1186/s12880-019-0342-4

**Published:** 2019-05-30

**Authors:** Eric Rydman, Sara Bankler, Sari Ponzer, Hans Järnbert-Pettersson

**Affiliations:** 1 Department of Clinical Science and Education, Södersjukhuset, Karolinska Institutet, Stockholm, Sweden; 2 Department of Radiology, Södersjukhuset, Stockholm, Sweden; 3 Department of Orthopedics, Södersjukhuset, SE-118 83 Stockholm, Sweden

**Keywords:** Disc degeneration, Neck pain -facet joint degeneration - computed tomography, Cervical spine, Scoring system

## Abstract

**Background:**

Grading of degeneration of the cervical spine is of great clinical value, considering the vast amount of radiological investigations that are being done with this query. Despite the fact that Computed Tomography (CT) is frequently used in clinical practice there is today no user-friendly and reliable scoring system for assessment of cervical spondylosis on CT-scans available. The aim of this study was to establish a scoring system for cervical spondylosis based on CT-scans and to test it for reliability.

**Methods:**

Twenty adult patients undergoing CT of the cervical spine due to neck pain following a motor vehicle accident were included in the study. Three independent raters, i.e. one orthopedic surgeon and two radiologists, assessed their CT-scans. Two of the raters repeated the assessments after three months. A radiographic-based scoring system for cervical disc degeneration, addressing disc height, osteophytes and endplate sclerosis, was applied on CT and tested for reliability. A pre-existing, reliable CT-based scoring system for facet joint degeneration, considering joint space narrowing, osteophytes and irregularity of the articular surface was modified and reevaluated. This in order to develop a coherent CT-based total degeneration score for cervical spondylosis.

**Results:**

The scoring systems for cervical disc degeneration and facet joint degeneration both exhibited an acceptable or better level of strength of agreement regarding intra- and interrater agreement. The total disc degeneration score showed a moderate level of inter-rater reliability with a kappa-value of 0.47 and a good intra-rater agreement with intra-class correlation coefficients (ICC) of 0.67 and 0.60 for the two raters performing the assessments. The total facet joint degeneration score showed a moderate level of inter-rater reliability (kappa 0.54) and an excellent intra-rater agreement with ICC 0.75 for one of the raters and fair for the other rater (ICC 0.54). When the total disc and facet joint degeneration score were classified into a three-point total degeneration score the inter-rater agreement was 0.695 and the ICC 0.82 and 0.73 respectively.

**Conclusions:**

This coherent scoring system assessing both disc degeneration and facet joint degeneration on CT-scans of the cervical spine was shown to meet the standards of reliability.

## Background

Cervical spondylosis is considered to be related to a complex process of pathophysiological and biomechanical factors [1–3]. It can often be asymptomatic and to date there is limited evidence that shows an association between subjective symptoms of unspecific neck pain and radiographic findings of spinal degeneration [4–6].

The discs and the facet joints are important for the biomechanical shift of stress on the cervical spine, as well as for mobility [1, 2]. The most common source of pain in post-traumatic chronic neck pain is suggested to be the facet joints [7]. However, other anatomical structures, including intervertebral discs may also be involved [8]. It is assumable that cervical spondylosis may have an adverse effect on the prognosis after neck trauma, although this has yet not been confirmed [9]. Previous studies investigating association between pre-existing cervical degeneration and outcome after trauma have based their assessments on plain radiographs or MRI with methods not tested for reliability [10–13].

Computed tomography (CT) scanning is a common method for detecting fractures and edema in an emergency department setting. Furthermore, CT is occasionally used in the medical investigations of patients with non-specific neck pain in general practice for detecting degenerative changes in intervertebral discs and facet joints. However, MRI is considered to be superior in evaluating degenerative changes in the cervical spine and is gold standard in evaluation of spondylosis [14]. The accuracy of detecting facet joint degeneration has contradicting been shown to be higher when using CT instead of MRI [15].

The radiological assessment of cervical spondylosis is often arbitrary regarding grading of degenerative findings. Reliable scoring systems already exist for degenerative changes in facet joints [16, 17]. However for degenerative discs there are so far only MRI-based [18] or radiographic-based scoring systems [19, 20]. With CT advancing its role over radiography in the diagnostics of acute spinal trauma [21] and its superiority in detecting degenerative changes [22], a coherent scoring system for both aspects of cervical spondylosis on CT is important. No such system is in broad clinical use today. Consequently, there is a lack of user-friendly and reliable scoring system based on CT-scans for coherent degeneration of the cervical spine.

The aim of this study was to establish an objective numerical scoring system for cervical spondylosis based on CT scans. The aims were to investigate whether a preexisting radiographic scoring system for cervical disc degeneration was applicable on CT and to reliability test the existing scoring system of cervical facet joint degeneration on CT. Both scoring systems have initially been developed by Walraevens et al. and exhibit satisfactory inter-rater agreement [16].

## Methods

**Fig. 1:**
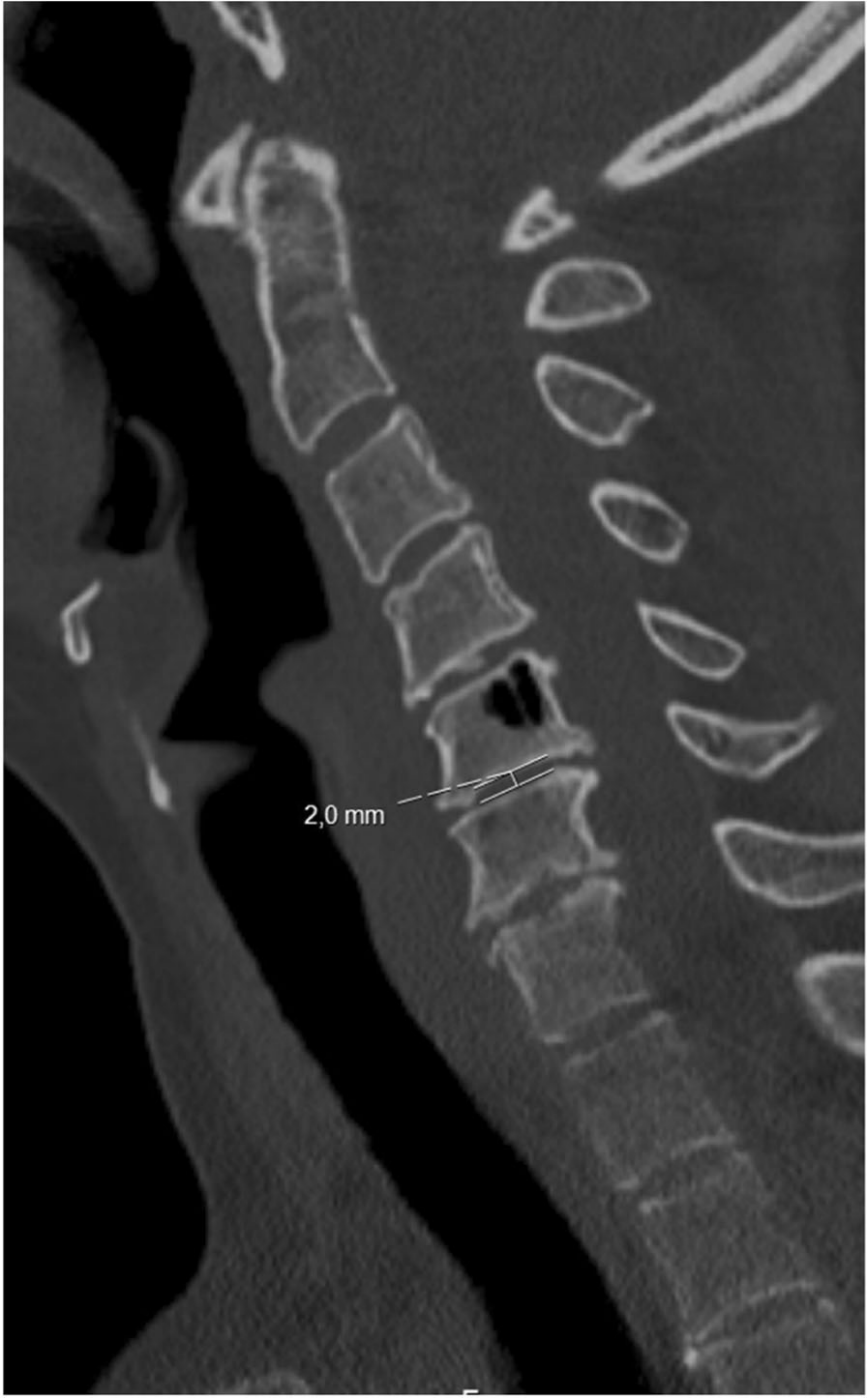
Disc degeneration exhibited by one of the study patients. Height loss of the C5-C6 disc, measuring 2 mm in a mid-sagittal slice. The height of a non-degenerated disc (C2-C3) was measured 4.2 mm, resulting in a relative height loss > 50%- ≤ 75% (3 points)

**Fig. 2:**
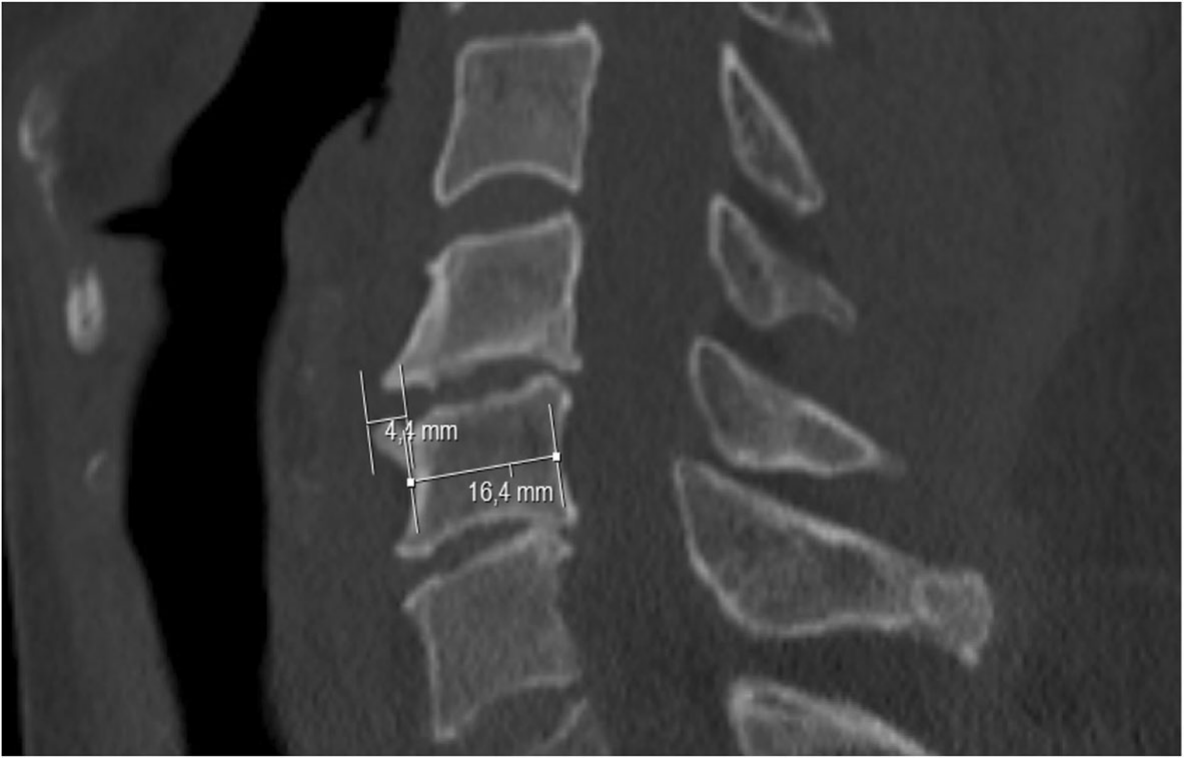
Most prominent anterior vertebral osteophyte. 4.4 mm/16.4 mm = 0.27= > 1/4 resulting in 3 points

**Fig. 3:**
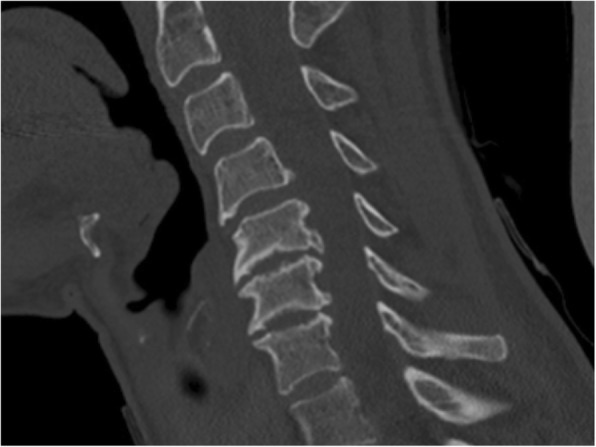
End plate sclerosis. Definite sclerosis on the end plates at the C5-C6-level resulting in 2 points

The scoring system for cervical disc degeneration used in this study is an adaption from an existing scoring system based on lateral radiographs. We used CT scans to determine the grade of degeneration. The scoring system consists of three variables: height loss, anterior osteophytes and endplate sclerosis (Table 1). As the degenerative process is suggested to begin with disc desiccation and height loss [1], this factor is ascribed the most importance and thus the largest impact on the total degeneration score. For all variables, the segment with the highest level of degeneration was chosen. Height loss was defined as the middle disc height measured in a mid-sagittal slice as compared to a normal (or least degenerated) disc height at any segment of the cervical spine as shown in Fig. 1. Anterior osteophytes were measured where the length was the greatest and compared to the anteroposterior diameter of the corresponding vertebral body as measured in the mid-sagittal slice (Fig. 2). Endplate sclerosis was ascribed on one of three discernible grades; no sclerosis, detectable sclerosis or definite sclerosis (Fig. 3). Finally, all variables were summed to an overall disc degeneration score (Table 1).

**Table 1:** Scoring system of cervical disc and facet joint degeneration. AP = anteroposterior

Disc degeneration		
*Height loss*	0%	0 points
	≤25%	1 point
	> 25- ≤50%	2 points
	> 50%- ≤ 75%	3 points
	> 75%	4 points
*Anterior osteophytes*	No osteophytes	0 points
	≤1/8 AP diameter	1 point
	> 1/8 - ≤1/4 AP diameter	2 points
	> 1/4 AP diameter	3 points
*Endplate sclerosis*	No sclerosis	0 points
	Detectable	1 point
	Definite	2 points
Overall degree of disc degeneration 1 + 2 + 3	0 points (no degeneration)	0 points
	1–3 points (mild degeneration)	1 point
	4–6 points (moderate degeneration)	2 points
	7–9 points (severe degeneration)	3 points
Facet joint degeneration		
*Joint space narrowing*	Normal	0 points
	Narrowed	1 point
*Osteophytes*	No osteophytes	0 points
	Yes	1 point
*Irregularity of articular surface*	Normal	0 points
	Irregular	1 point
Overall degree of facet joint degeneration (1 + 2 + 3)	0 points (no degeneration)	0 points
	1 point (mild degeneration)	1 point
	2 points (moderate degeneration)	2 points
	3 points (severe degeneration)	3 points

**Fig. 4:**
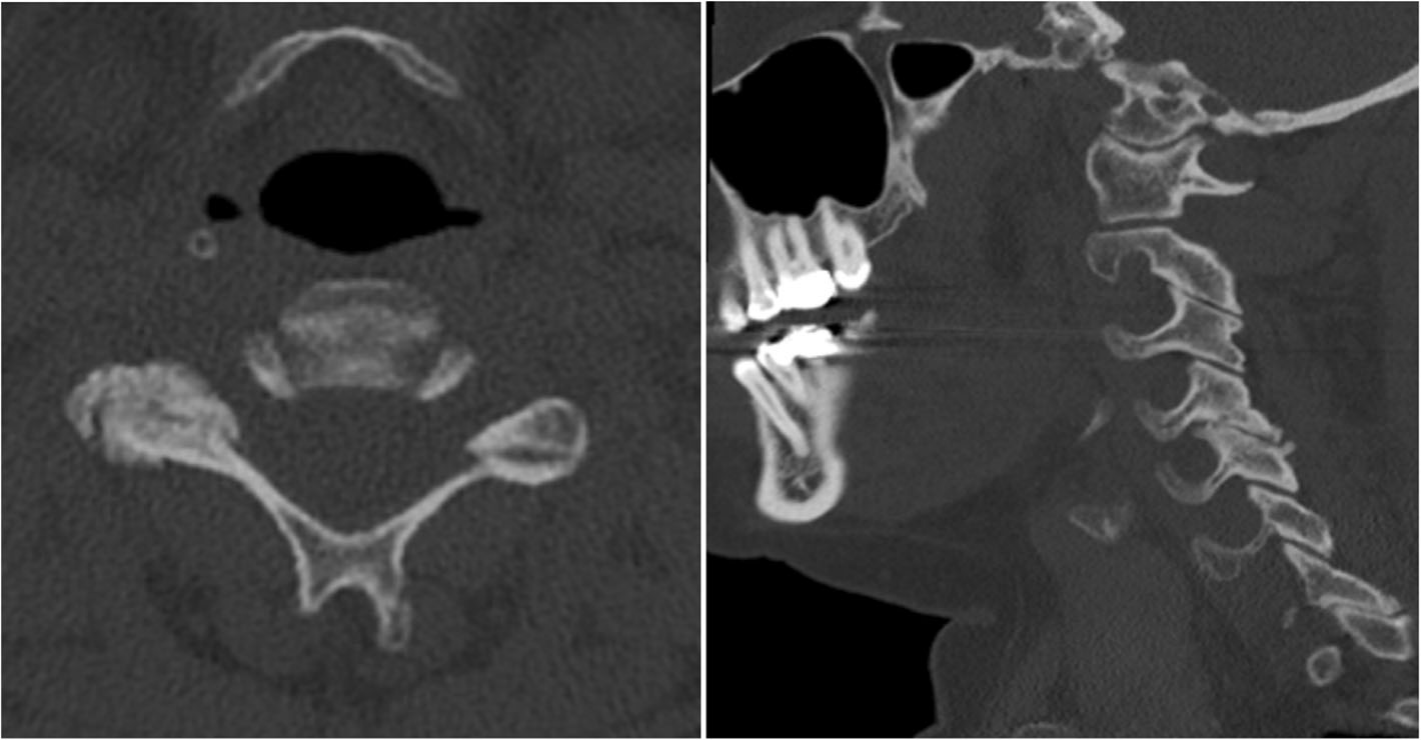
Facet joint degeneration as exhibited by one of the study patients. To the left, an axial slice through the C5-C6 segment shows joint space narrowing, osteophytes and marked irregularities of the right facet joint space leading to 3 points. The right image illustrates the facet joint space narrowing at the same segment as compared to normal joint spaces on adjacent levels on a lateral view

**Fig. 5:**
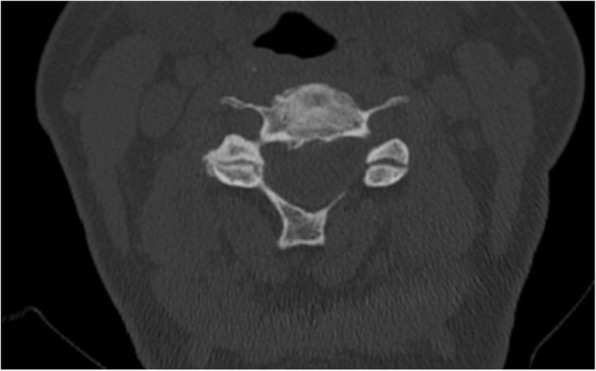
Facet joint with pronounced joint space narrowing and ostephytosis. However, no obvious irregularities are present. 2 points

The scoring system for cervical facet joint degeneration was developed for CT. It contains three variables: joint space narrowing, osteophytes and irregularity of the articular surface (Table 1). The original scoring system [16] also included facet joint hypertrophy, which we chose to exclude as it was the variable with the lowest inter-rater agreement in the previous study and offered limited additional information on the degree of facet joint degeneration. Joint space narrowing was assessed on sagittal scans and if any of the facet joints were narrowed, this variable was ascribed as 1 point (Fig. 4). Osteophytes and joint space irregularity were assessed on axial scans. If any osteophytes were present, the variable was ascribed as 1 point. Similarly, the presence of joint space irregularity received 1 point whereas smooth articular surfaces received 0 points (Fig. 5). Finally, the variables were summed to achieve an overall facet joint degeneration score.

For the total degeneration score, all variables were summed up to achieve an overall degeneration score for cervical spondylosis and were categorized as 0–1 p – no degeneration, 2–6 p – moderate degeneration and 7–12 p – severe degeneration.

### Procedure

Patients were examined in a Philips Brilliance 64-slice CT scanner. A special study protocol was designed since the patients included were also eligible to participate in a related study at the same institution. The study CT protocol was extended craniocaudally to include the clivus and sternal tip. The expanded FOV was compensated with a low radiation dose profile with CTDI_vol_ of around 3.8.

Patients aged > 18 years that were admitted to the emergency department at Södersjukhuset, Stockholm, Sweden for neck pain after a motor vehicle accident were included in the study. Those requiring medical imaging in the emergency setting according to the Canadian C-spine rules [23] underwent a CT of the cervical spine. The patients whom the examining physician deemed not to require medical imaging were later contacted by the research team and offered to participate in the study. If they accepted, they were also examined with a CT of the cervical spine. All patients had been contacted and gave their informed consent prior to the CT scan.

### Statistical analysis

The statistical software package SPSS 22 (SPSS Inc., Chicago, IL) was used for analysis of both intra-rater and inter-rater agreement. An additional SPSS macro was used to compute the inter-rater agreement level for categorical data between multiple observers [24]. The Kappa values were considered significant if *p* < 0.05. The intra-rater agreement was assessed with intra-class correlation coefficient (ICC).

### Interpretation

The interpretation guideline established by Landis & Koch [25] was used to evaluate the strength of inter-observer agreement using the kappa statistic.

Kappa values of > 0.40 were considered representing clinically acceptable level of strength of agreement for a scoring system [26].

The standards for strength of intra-rater agreement proposed by Fleiss et al. [27] were used as a basis for interpretation of the magnitude of the ICC values obtained.

## Results

### Inter-rater reliability

**Fig. 6:**
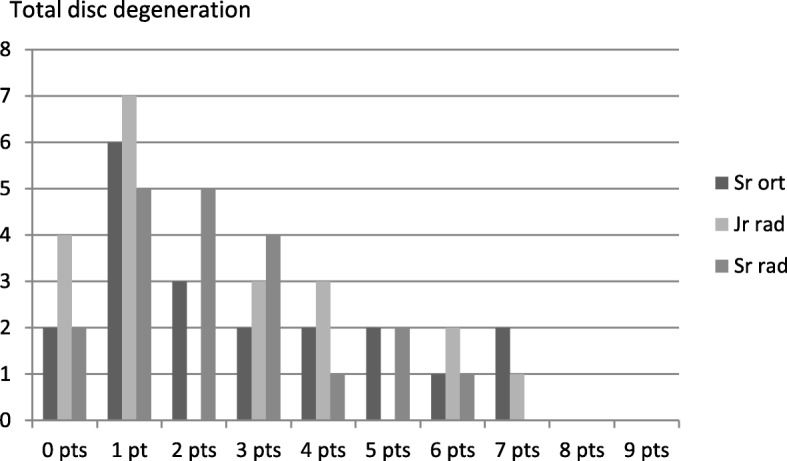
Total disc degeneration score (0–9 points) as assessed by the three raters; the senior orthopedic surgeon (Sr ort), junior radiologist (Jr rad) and the senior radiologist (Sr rad) respectively

**Fig. 7:**
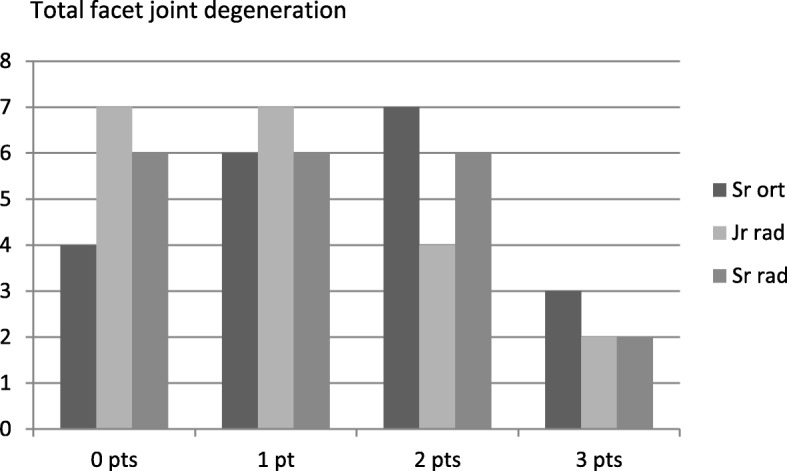
Total facet joint degeneration score (0–3 points) as assessed by the three raters

**Fig. 8:**
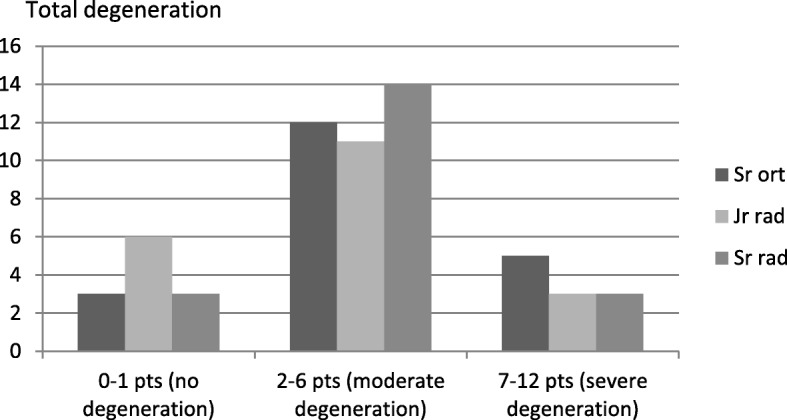
Total degeneration score (0–12 points) as assessed by the three raters

Figures 6, 7 and 8 illustrate the variance in degeneration scores as assessed by the three raters regarding discs, facet joints and total degeneration, respectively (Figs. 6, 7 and 8).

As shown in Figs. 6, 7 and 8, the senior orthopedic surgeon had a consistent tendency to assign the variables a more severe degree of degeneration than both the radiologists. A statistical analysis of the agreement in scorings by the three raters generated kappa values for the variables (Table 2). Anterior osteophytes generated the highest kappa value, i.e. the raters tended to rate the presence and size of anterior osteophytes similarly. Regarding facet joint degeneration, in terms of joint space narrowing the raters were closest to agreement.

**Table 2:** Inter-rater agreement between the three raters regarding the degree of degeneration

Disc degeneration		
Variable	Kappa value	*p*-value
Height loss	0.467	< 0.001
Anterior osteophytes	0.633	0.000
Endplate sclerosis	0.241	0.092
Overall degree of disc degeneration (score 0–9 points)	0.470	< 0.001
Facet joint degeneration		
Variable	Kappa value	p-value
Joint space narrowing	0.569	< 0.001
Osteophytes	0.313	0.154
Irregularity of articular surface	0.365	0.011
Overall degree of facet joint degeneration (score 0–3 points)	0.542	< 0.001
Total degeneration		
Variable	Kappa value	p-value
Total degeneration score (0–12 points)	0.695	< 0.001

### Intra-rater reliability

To test the intra-rater reliability and thus reproducibility of the scoring system, the senior orthopedic surgeon and the junior radiologist repeated their assessments of the 20 patients after a minimum of 3 months (Table 3). The analysis showed excellent agreement between the two assessments for the total degeneration and at least fair agreement for all parameters with ICC spanning from 0.538 to 0.820.

**Table 3:** Intra-rater agreement between the two assessments made by the junior radiologist and the senior orthopedic surgeon, respectively

Intra-rater agreement between the two assessments				
	Junior radiologist		Senior orthopedic surgeon	
	ICC	95% CI	ICC	95% CI
Disc degeneration	0.672	0.103–0.875	0.598	0.023–0.838
Facet joint degeneration	0.538	−0.284-0.833	0.750	0.361–0.902
Total degeneration	0.820	0.542–0.929	0.727	0.076–0.905

*ICC* intraclass correlation coefficient, *CI* confidence interval

## Discussion

Despite the vast amount of CT-scans of the cervical spine that are made addressing degenerative changes, to date there is a lack of clinical standardized rating models. Cervical spondylosis is a common radiological finding and the association to disability and pain is still unclear. This might be partly due to lack of consensus in grading models for degeneration why it is of importance to obtain reliable assessment models. The effort of this study was to contribute to establishing such a scoring system and validate it in the aspects of inter-rater and intra-rater reliability. Focus was put in creating a user-friendly system for clinical implementation.

### Inter-rater reliability

The kappa value for the overall degree of degeneration showed a substantial agreement. However, this value represents the agreement between the raters when adding their degeneration scores on the separate variables and then dividing the subjects into three separate categories (no degeneration, moderate degeneration or severe degeneration). When grouped together, the rate of disagreement on the separate variables is masked and the agreement when only three categories exist is presumably consequently higher than it would be if more than three categories of degeneration were eligible. This becomes apparent when analyzing the separate variables, where kappa values are considerably lower. In two of the variables the null-hypothesis could not be rejected (endplate sclerosis and facet joint osteophytes). The only variable where the strength of agreement was substantial was anterior osteophytes. This variable is weighted to contribute less to the disc degeneration score than height loss, which only reached a moderate strength of agreement. The fact that agreement for the total level of degeneration was higher than for the separate variable could be explained by compensation mechanisms of the individual rater. For example, a borderline case of facet joint osteophytes could have been neglected with a compensatory affirmation of borderline irregularity of the articular surface.

Our study showed similar results with those of the study of Walraevens et al. [16] concerning facet joint degeneration, even though our classification criteria differed, with low strength of agreement on osteophytes and irregularity of the articular surface but slightly higher strength of agreement for joint space narrowing.

However, when applying the radiograph-based scoring system for disc degeneration to CT there seemed to have been a slight loss of reliability compared to Walraevens et al. [16]. They showed “good” or “excellent” agreement on the disc degeneration variables apart from endplate sclerosis which was low in both studies, whereas our results ranged from “moderate” to “substantial” with a slightly lower level of agreement overall. However, the trend is clear; assessing endplate sclerosis, facet joint osteophytosis and irregularity of facet joint articular surfaces is more complex than the three other variables.

Considering a cut-off limit of 0.40 for strength of agreement, which is arbitrarily set, many of our obtained kappa values indicate an acceptable or good level of agreement. However, several Kappa values were below 0.40. There are a few reasons for the relatively low values that must be considered. First, the relatively small sample size could have affected the level of agreement. Another factor might have been the multi-segment assessment. Determining the spinal segment with the highest level of degeneration is an assessment by itself. It is plausible that the raters were in fact reviewing different segments and consequently assessing them differently. Lack of training among the raters might also affect the level of agreement. In this material, the raters deliberately had no joint training session of the scoring system prior to the assessment procedure. This was to simulate a clinical setting to a high extent.

The goal of developing a scoring system that is easily applicable and experience- and discipline independent is of importance. However, we believe minor modifications could be done to improve the scoring system while still keeping it user-friendly. For example, one source of disagreement on the height loss-variable may have been presence of endplate compression affecting the disc height.

### Intra-rater reliability

The ICC-values obtained all indicated fair, good or excellent intra-rater agreement, with total degeneration scores having the strongest agreement for both raters. However, the confidence intervals were large and the true ICC-values thus hard to discern. They are interpreted to originate from the variation between examiners using an ordinal scale on a relatively small material. Only two of the raters participated in the intra-rater reliability part of the study. As in the inter-rater analysis, the agreement of the total degeneration score was higher when summing disc degeneration and facet joint degeneration scores.

In comparison with other scoring scales in the field the agreement is regarded equivalent. Considering inter-rater reliability of the assessment of disc degeneration, previous scales vary from 0.41–0.78 [16, 28, 29] intra-rater reliability of the discs vary from 0.71–0.86 [16, 29]. In the material reviewed, the inter-rater agreement for facet joint degeneration the agreement varied from 0.43–0.49 [15, 16] and the intra-rater agreement from 0.57–0.72 [15, 16]. When comparison is made, one must consider the different radiologic modalities that are used in previous materials.

In summary, our results indicate a well acceptable level of agreement regarding both inter-rater and intra-rater reliability of a CT based scoring system, especially addressing facet joint degeneration and overall degeneration. The findings enable a role for this scoring system in both future research and clinical practice. However, when analyzing individual parameters in the scores, the agreements were lower than in the total scores. Hence, we recommend the system to be clinically applied in its aggregated form to assess disc degeneration, facet joint degeneration and overall degeneration.

This study has a few limitations. First, the sample size is rather small and for wide clinical implication, future studies with larger material are required to confirm the results.

Second, the study population in this this material consists exclusively of post-traumatic patients. This makes it less representable for the general population and is neither to be considered an asymptomatic cohort nor a cohort with non-specific neck pain. We welcome further investigations in a different clinical setting to validate the scoring system.

## Conclusions

To our knowledge, this is the first study evaluating a coherent scoring system for degeneration of the cervical spine based on CT. It confirms that a preexisting scoring system for cervical facet joint degeneration has an acceptable level of strength of agreement for the overall degeneration. This study also showed that a radiographic scoring system for cervical disc degeneration is applicable on CT, achieving a moderate degree of strength of agreement for the overall degeneration. Both scoring systems meet the standards for a clinically accepted level. Combined or individually, they make a reliable, coherent and objective scoring system readily applicable in both research and in clinical settings where it can simplify and objectify the assessment of presence and degree of cervical degeneration.

## Data Availability

The data and materials in this article can be made available upon request by sending an e-mail to the corresponding author.

## Abbreviations

CT: Computed Tomography
CTDIvol: Volume Computed Tomography Dose Index
FOV: Field of View
ICC: Intraclass Correlation Coefficient
